# An ectomycorrhizal fungus alters developmental progression during endogenous rhythmic growth in pedunculate oak

**DOI:** 10.1007/s00572-025-01228-1

**Published:** 2025-10-11

**Authors:** Felix Zimmermann, Marie-Lara Bouffaud, Sylvie Herrmann, Marco Göttig, René Graf, Mika Tarkka, Lars Opgenoorth, Daniel Croll, Martina Peter, Benjamin Dauphin

**Affiliations:** 1 Biodiversity and Conservation Biology, Swiss Federal Research Institute WSL, Birmensdorf, Switzerland; 2 Laboratory of Evolutionary Genetics, Institute of Biology, University of Neuchâtel, Neuchâtel, Switzerland; 3 Department of Soil Ecology, Helmholtz Centre for Environmental Research - UFZ, Halle/Saale, Germany; 4 German Centre for Integrative Biodiversity Research (iDiv) Halle-Jena- Leipzig, Leipzig, Germany; 5 Plant Ecology and Geobotany, Institute of Biology, Philipps University Marburg, Marburg, Germany

**Keywords:** *Cenococcum geophilum*, Ectomycorrhiza, Endogenous rhythmic growth, *Piloderma croceum*, *Quercus Robur*

## Abstract

Pedunculate oak (*Quercus robur* L.), a long-lived forest tree species, forms symbiotic relationships with ectomycorrhizal (ECM) fungi, which can promote nutrient uptake, stress resilience, and growth. Like other tropical and temperate tree species, pedunculate oak exhibits endogenous rhythmic growth (ERG), a trait conferring the ability to repeatedly alternate root and shoot flushes as well as growth cessation as response to changing environmental conditions. However, the effects of different ECM fungal species on the ERG dynamics remain largely unknown. Here, we investigated the impact of two ECM fungi—*Piloderma croceum*, a basidiomycete previously shown to promote growth while not found in natural oak stands, and *Cenococcum geophilum*, an oak-native ascomycete with broad ecological range—on growth performance, biomass partitioning, and ERG patterns in a clonal oak system (clone DF159). By combining in vitro experiments with Bayesian modelling, we show that *P. croceum* promotes tree growth among treatments, without disrupting the endogenous growth rhythm. In contrast, *C. geophilum*, while showing high mycorrhization rates, led to reduced biomass accumulation and altered developmental progression through the ERG stages, especially by prolonging the steady state development stage—part of the root flush and characterized by peak net carbon assimilation. Co-inoculation revealed a competitive advantage of *C. geophilum* in root colonization, yet growth responses resembled those of the control. Our findings demonstrate that ECM species exert species-specific effects on biomass production and temporal development of plants, underscoring the functional importance of ECM fungi in shaping host development. Assessing these interactions provides new insights into the functional diversity of ectomycorrhizal symbiosis and can inform forest management strategies aimed at enhanced resilience in oak-dominated ecosystems under rapidly changing climatic conditions.

**Supplementary Information:**

The online version contains supplementary material available at 10.1007/s00572-025-01228-1.

## Introduction

Pedunculate oak (*Quercus robur* L.) has been ecologically and economically valued in Europe for centuries (Eaton et al., 2016; Haneca et al. 2009). As a widely distributed and long-lived forest tree species, *Q. robur* forms diverse ecological interactions, notably with a broad spectrum of microorganisms (Jumpponen and Jones 2009), including ectomycorrhizal (ECM) fungi. These interactions commonly result in the formation of ECM root tips, which play a critical role in plant nutrient acquisition, water uptake, and stress tolerance (Herrmann and Buscot 2007; Richard et al. 2005). To better understand these biotic interactions, a genetically uniform clonal oak system (oak clone DF159) has been established and serves as a valuable model for studying both biotic and abiotic influences on *Q. robur*, with a particular emphasis on symbiosis with ECM fungi under experimental conditions (Bouffaud et al. 2020; Herrmann et al. 1998, 2004; Tarkka et al. 2013).

*Quercus robur* is of particular interest as the species exhibits endogenous rhythmic growth (ERG), a trait shared by various tropical and temperate forest tree species (Barthélémy and Caraglio 2007). The ERG trait allows trees to adjust growth dynamics to fluctuating environmental conditions throughout a growing season (Borchert 1975). Thus, *Q. robur* is showing alternating root and shoot flushes which may be subject to growth cessation under unfavorable conditions, i.e. sudden temperature changes and drought events (Herrmann et al. 2015). Importantly, ERG has been shown to be regulated internally, i.e. through endogenous clocks, rather than directly by external resource availability (Herrmann et al. 2015). Herrmann et al. (2015) identified several genes involved in the circadian clock gene network to be associated with ERG, though the role of circadian clock genes in the ERG of *Q. robur* is not yet fully resolved. The circadian clock, i.e. the 24-hour rhythm in metabolic, physiological, and behavioral patterns of eukaryotes (Millar 2016), affects at least 30% of transcripts in *Arabidopsis thaliana* and therefore plays a major role in gene regulation processes (Covington et al. 2008). Furthermore, the circadian rhythm of plant species confers a photosynthetic and competitive advantage while facilitating increased growth- and survival-rates (Dodd et al. 2005). The previously mentioned gene regulation processes are triggered by changes in environmental conditions that are perceived by various specific receptors, allowing plants to respond to environmental variation and consequently adjust their metabolism (McClung 2001; Singh et al. 2021). In *Q. robur*, ERG has been linked to key physiological processes such as growth coordination and defense priming (Fernández et al. 2024). Recent findings suggest that while ERG is genetically programmed, its resource allocation and biomass partitioning dynamics can be modulated by biotic factors, and that these changes may be enhanced by mycorrhizal associations (Tarkka et al. 2021). However, whether these interactions with ECM fungi can influence the duration of developmental stages during the ERG itself—beyond growth-stimulation and shifts in biomass partitioning—remains unexplored.

To investigate this, we focus on two ECM fungal species with contrasting evolutionary histories, ecological traits, host preferences and habitat-ranges. First, we used *Cenococcum geophilum*, a cosmopolitan ascomycete naturally associated with oaks, known for its wide ecological niche and pioneering role in root colonization (Peter et al. 2016; Wang et al. 2025). Second, we considered *Piloderma croceum*, a basidiomycete naturally associated to beech (*Fagus sylvatica*; Goldmann et al. 2015) while not yet found in natural oak stands. This species has been shown to enhance biomass production in the DF159 oak clone by improving resource availability at the rhizosphere level, though without altering ERG patterns (Herrmann et al. 2015). Despite the native status and ecological relevance of *C. geophilum*, its effects on oak growth performance and the dynamics of ERG have not yet been tested in the in vitro DF159 clone system. To fill this knowledge gap, we addressed the following research questions: (i) How do different ECM fungi affect biomass production and partitioning in the oak clone DF159? (ii) Does a native ECM fungus outcompete another ECM fungus that has not yet been found in natural oak stands after co-inoculation? (iii) Can biotic interactions with ECM fungi modulate the dynamics of ERG development stages and growth cycles?

## Materials and methods

### Experimental setup and conditions

We micropropagated and rooted the pedunculate oak (*Quercus robur* L.) clone DF159 according to Herrmann et al. (1998). In brief, we alternatively used micro-cuttings of axillary and apical buds to maintain the ERG of oak plants (Herrmann et al. 2016). After micropropagation under sterile conditions in glass vessels, we transferred individual cuttings to sterile glass tubes containing active charcoal medium for in vitro rooting. We performed both the micropropagation and in vitro rooting at 24 °C, 50% air humidity, and under long-day conditions (16 h of light per day) with a photosynthetic photon flux density of 90–100 µmol m^−2^ s^−1^.

After plant production, we prepared the experimental culture system according to Tarkka et al. (2013). In short, we sieved at 2 mm soil originating from an oak forest located in the Harz mountains, Saxony Anhalt, Germany (51.400972, 11.125389), collecting the A0 (humus, 0–10 cm) and A1/A2 (organic, 10–30 cm) horizons, and sterilized the sieved fraction at Synergy Health Däniken AG (Däniken, Switzerland) using γ-irradiation with a dosage of 70–90 kGy. We cultivated *Piloderma croceum* strain F1598 (ATCC MYA-4870; Herrmann et al. 1998, 2015) and *Cenococcum geophilum* strain 1.58 (CBS 143616; Peter et al. 2016) on Modified Melin-Norkrans (MMN; Marx 1969) agar medium in darkness at 20 °C (Herrmann et al. 1998). We prepared MMN liquid cultures using plugs collected from mycelium grown on agar plates. Finally, we transferred the liquid cultures onto 10:1 (v/v) mixtures of vermiculite and peat (autoclaved three times) and incubated them in darkness at 20 °C for four weeks.

**Fig. 1:**
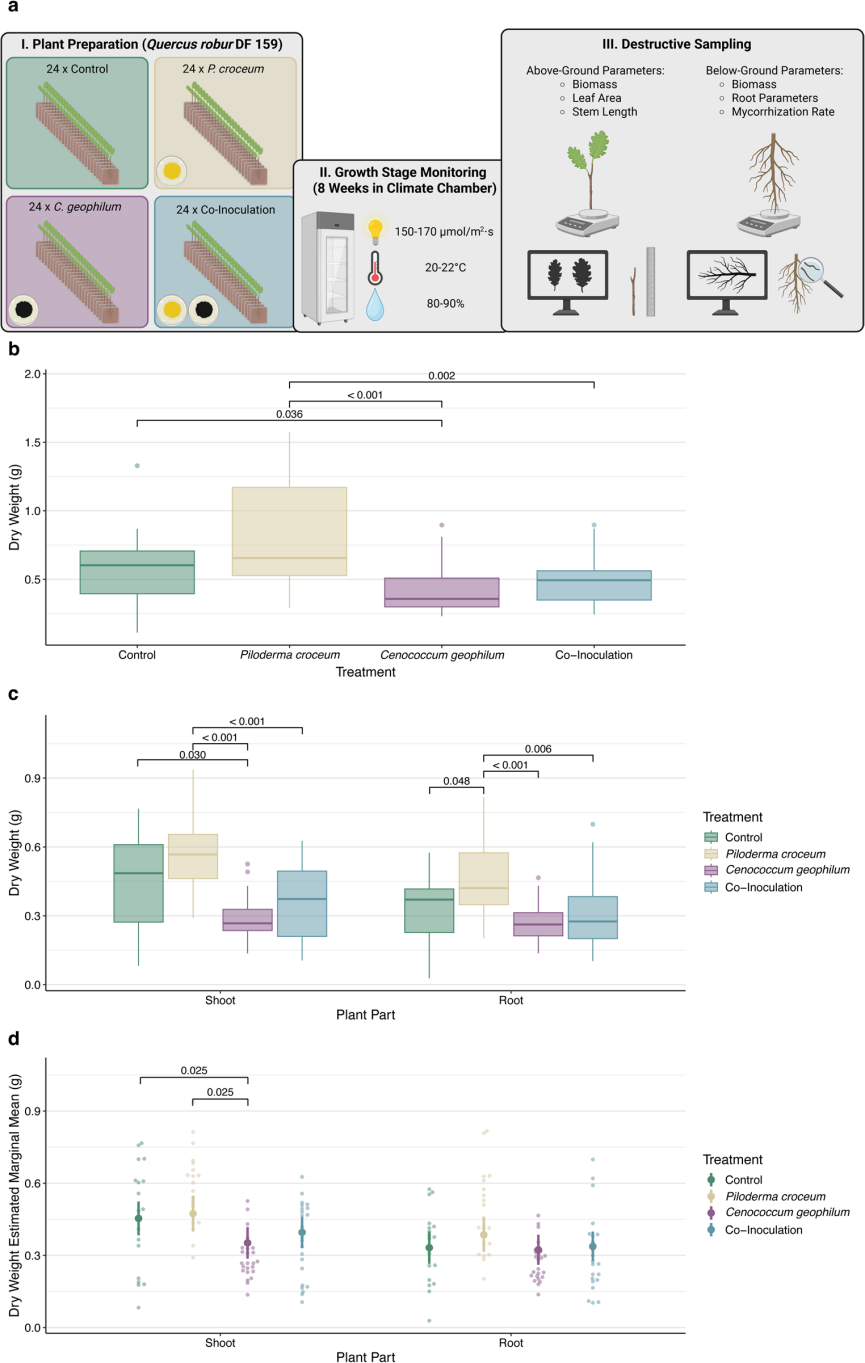
Overview of the experimental setup and response of phenotypic traits.** a** Illustrates experimental setup and key working steps (created with BioRender). **b** Shows raw total plant dry weight (y-axis) colored and arranged on the x-axis by treatment. Overhead lines connecting boxplot pairs indicate *p*-values of significant pairwise Wilcoxon tests. **c** Represents dry weight (y-axis) of plant root and shoot (x-axis) colored by treatment. Overhead lines connecting boxplot pairs show *p*-values of significant pairwise Wilcoxon tests. **d** Indicates the estimated marginal means of root and shoot (x-axis) dry weight (y-axis) colored by treatment after controlling for total plant dry biomass in a generalized linear model (plant part dry weight ~ treatment + plant dry weight) using a Gaussian distribution family. Lines represent 95% confidence intervals of estimated marginal means and less saturated points represent the original data. Overhead lines connecting treatments show significant *p*-values of the model-based pairwise z-test

We then applied four treatments to the clone after randomizing the plants according to development stage, rooting date and stem length: the control treatment (Table S1), the *C. geophilum* treatment, the *P. croceum* treatment, and the co-inoculation treatment. To do so, we placed each of the oak plants in a 12 × 12 cm squared Petri dish (roots grown inside, shoots grown outside the Petri dish), assessed the initial weight and sealed the Petri dish with parafilm. In total, we prepared two sets of 48 plants each (with 12 plants per treatment, Fig. 1a), with the second set being produced three weeks after the first set. We grew the plants in a growth chamber at 20–22 °C, 80–90% air humidity, and with 16 h of constant light as previously described. All data generated during the experiment were stored according to the FAIR (Findable, Accessible, Interoperable, and Reusable) data principles (Weil et al. 2023).

## Monitoring of development stages and growth cycles

**Fig. 2:**
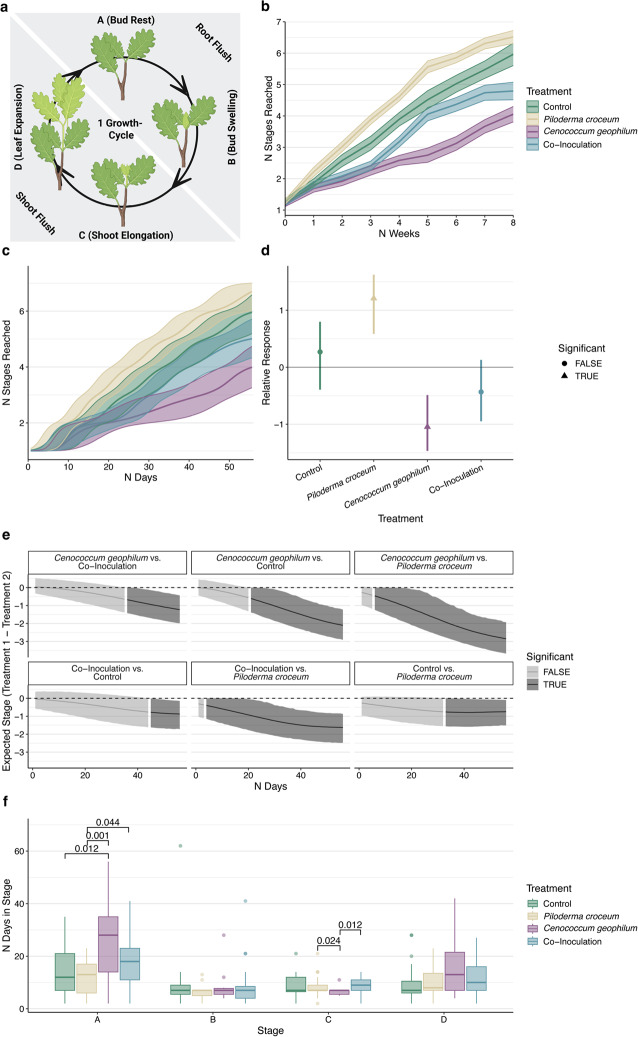
Temporal dynamics of development stages and growth cycles across treatments.** a** Illustration of the endogenous rhythmic growth exhibited by *Quercus robur* according to Herrmann et al. 2015 (created with BioRender). Stages A (bud rest) and B (bud swelling) represent the root flush, while stages C (shoot elongation) and D (leaf expansion) correspond to the shoot flush. **b** In-vitro observed development stages over time, colored by treatments. Experimental weeks are represented on the x-axis, reached development stages are represented on the y-axis. Thick lines represent the mean, shaded areas the standard errors. **c** Modelled development stage progression based on Bayesian regression using a cumulative distribution family. The x-axis shows modelled days; the y-axis shows predicted stages reached. Thick lines represent model estimates, shaded areas 95% credible intervals. **d** Overall treatment effects relative to the overall mean colored by treatments. Treatments are represented on the x-axis, relative treatment effects (estimates) on the y-axis. Thick lines represent model estimates, shaded areas 95% credible intervals. Point shapes indicate significance. **e** Pairwise comparisons of treatment effects over time. Modelled days are represented on the x-axis, Delta of modelled stages reached (treatment 1 – treatment 2) are represented on the y-axis. Gray tones represent significance of difference in pairwise treatment effects (95% credible intervals for treatment difference exclude 0). **f** Time spent in individual developmental stages A–D (according to Herrmann et al. 2015), colored by treatment. The x-axis shows development stages; the y-axis indicates days spent in each stage. Wilcoxon rank sum tests were used to assess treatment differences. Overhead lines connecting boxplot pairs show *p*-values of significant pairwise Wilcoxon tests

Day 1 of the experiment is defined as the first day after a four-week acclimatization period in protective plastic bags. Throughout eight weeks of the experiment, we assessed the development stages of the plants twice a week according to Herrmann et al. (2015; Fig. 2a). In short, *Q. robur* growth was characterized by the succession of developmental stages A (bud rest) and B (bud swelling), corresponding to the root flush of a growth cycle, followed by stages C (shoot elongation) and D (leaf expansion), corresponding to the shoot flush. We weighed the plants weekly and replenished any weight loss with sterilized tap water to maintain optimal growth conditions at a soil water content of 12–15%.

## Above and belowground destructive sampling

After 64 days (set one) and 58 days (set two) of cultivation, we destructively sampled all above- and belowground parts of the plants (Table S2). We measured total plant biomass, followed by a separate weighing of above and below ground plant parts. We further divided above ground biomass into stem, leaves from the newest shoot flush, leaves from the second newest shoot flush, remaining leaves, and buds. We counted leaves from the last two shoot flushes and scanned them using an EPSON Perfection V700 Photo scanner (SEIKO Epson CORPORATION, Tokyo, Japan) to determine leaf area. The root system was scanned with the same EPSON Perfection V700 Photo scanner and analyzed with WinRhizo 2003b (Regent Instruments, Québec, Canada) to quantify root parameters; total root length, total root surface area, average root diameter, total root volume, number of root tips, number of root forks, number of root crossings, length of fine roots (0–1 mm diameter), surface area of fine roots (0–1 mm diameter), and volume of fine roots (0–1 mm diameter). We separated and weighed principal and lateral roots; the latter defined as diverging more than 1 cm from the principal roots. To assess mycorrhization rate, we examined root tips from ~ 10% of the lateral roots for colonization by *C. geophilum* and *P. croceum* versus non-mycorrhized tips using the gridline intersection method (Phillips and Hayman 1970), which we modified by focusing on ectomycorrhizal colonization. Instead of scoring intraradical fungal structures, we counted each root tip intersecting the grid as either colonized (i.e., visibly forming a mantle) or non-colonized. Finally, we dried all plant materials at 55 °C for two weeks and measured dry weights.

## Phenotypic trait response analysis

We excluded dead and contaminated plants from all analyses. To test for significant differences in total biomass (dry weight), we performed non-parametric Wilcoxon rank sum tests using R (v4.4.1, R Core Team 2024) to account for non-normality of the data (visually assessed using Q-Q plots) and applied Benjamini-Hochberg corrections for multiple comparisons (Benjamini and Hochberg 1995). We used the same approach for pairwise comparisons of shoot and root dry weights. To assess treatment effects on root and shoot biomass partitioning independently of total biomass, we fitted generalized linear models (Gaussian distribution) with treatment and total plant dry weight as predictors. We then calculated estimated marginal means (EMMs) using the emmeans function from the emmeans R package (Lenth 2024), as it allows for comparisons between treatment groups while controlling for the effects of total plant biomass, thereby providing a measure of treatment-specific effects on shoot and root biomass partitioning. Based on the EMMs, we conducted a model-based pairwise z-test using the pairs function of the emmeans R-package (Lenth 2024). Here, we used a parametric test since an appropriate distribution family was used in the generalized linear model and applied a Benjamini-Hochberg correction to account for multiple testing (Benjamini and Hochberg 1995).

## Time series analysis of development stages and growth cycles

To visualize development progression between treatments, we plotted the number of development stages reached during the experiment (week 0 to 8; week 8 was the last with biweekly stage assessments for both plant sets). A biweekly stage assessment was chosen based on a previous study that demonstrated this timing efficiently captures developmental transitions in the DF159 oak clone (Herrmann et al. 2015). The beginning stage at the start of the experiment was considered as the first stage reached by a plant. We then fitted a Bayesian regression model using the brm function from the brms R package (Bürkner 2017) with 6,000 iterations (3,000 burn-in) across four chains with different levels of complexity. We selected the best model fit for further analysis based on Bayesian *R*^2^ values, R-hat convergence diagnostics, bulk effective sample sizes (ESS) and leave-one-out information criterion (LOOIC; Gabry et al. 2019) using the Stan R-interface (Stan Development Team 2025). In the final model, we used number of stages reached as response variable with treatment as fixed effect while including random intercepts and slopes for individual trees to account for plant-specific growth variations. Furthermore, we used a spline-function to allow for treatment-specific non-linear growth patterns (Wood et al. 2016). To reflect the cumulative but non-uniform nature of stage development, the cumulative distribution family was applied (Bürkner 2017). We used the conditional_effects function to generate predicted stage development trajectories and infer treatment effects which were considered significant if Bayesian 95% credible intervals excluded zero. We then used the posterior_epred function to model treatment-specific predictions over the entire eight-week period (days 1–56). For each day, we performed pairwise comparisons between treatments and determined the time point at which differences became significant, based on non-overlapping 95% credible intervals. Finally, we applied a non-parametric Wilcoxon rank sum test to assess differences in non-normally distributed durations that plants of different treatments took to first, complete a whole growth cycle and second, numbers of days those plants spent in each of the four development stages (A–D). In both cases, we used the Benjamini-Hochberg correction for multiple comparisons (Benjamini and Hochberg 1995).

## Results

### Phenotypic trait response analysis

The experiment achieved a high plant survival rate of 97% (three dead plants) and a low contamination rate of 7% (six contaminated plants) which was homogeneous across randomized plant sets (Fig. S1). After excluding dead and contaminated plants, 87 out of 96 trees were retained for further analysis. Based on a chi-square test, none of the treatments had a significantly higher or lower number of excluded (*p* = 0.06). Pairwise Spearman correlations of phenotypic traits are shown in Fig. S2. Among treatments, *Piloderma croceum*-inoculated plants produced the highest mean total biomass (dry weight = 0.82 g, coefficient of variation [CV] = 0.45; Fig. 1b), with a mean mycorrhization percentage of 1.87% (CV = 1.2). Control plants followed with a dry weight of 0.58 g (CV = 0.47) and mycorrhization percentage of 0%. The co-inoculated plants had a mean dry weight of 0.49 g (CV = 0.38), with 9.7% of root tips (CV = 0.94) colonized by *Cenococcum geophilum* and none by *P. croceum*. *Cenococcum geophilum*-inoculated plants accumulated the lowest biomass (0.42 g, CV = 0.41), while showing the highest mycorrhization percentage (17%, CV = 1.03). Wilcoxon tests revealed that *P. croceum*-inoculated plants had significantly higher total dry weight compared to *C. geophilum-* (*p* < 0.001) and co-inoculated plants (*p* = 0.002; Fig. 1c). This pattern was consistent for shoot and root dry biomass. Control plants had significantly higher total plant dry weights compared to *C. geophilum*-inoculated plants (*p* = 0.036), which was consistent for shoot dry weight. *P. croceum*-inoculated plants exhibit the highest mean shoot dry weight (0.58 g, CV = 0.27; Fig. 1c), followed by control (0.46 g, CV = 0.47), co-inoculation (0.36 g, CV = 0.43), and *C. geophilum* treatments (0.29 g, CV = 0.32). Pairwise comparisons confirmed significantly higher shoot dry biomass in *P. croceum*-inoculated plants compared to *C. geophilum* (*p* < 0.001) and co-inoculation treatments (*p* = 0.012), while control plants had accumulated significantly higher shoot dry weight than *C. geophilum*-inoculated ones (*p* = 0.030). Similarly, the *P. croceum*-inoculated plants had the highest mean root dry weight (0.47 g, CV = 0.36), followed by control (0.36 g, CV = 0.44), co-inoculation (0.31 g, CV = 0.52), and *C. geophilum* treatments (0.27 g, CV = 0.31). As for the total and shoot dry biomass, *P. croceum*-inoculated plants showed significantly higher root dry biomass than *C. geophilum*- (*p* < 0.01) and co-inoculated plants (*p* < 0.01), and in addition also compared to control plants (*p* = 0.048).

When controlling for total plant dry weight, estimated marginal means (EMM) showed that *P. croceum*-inoculated plants partitioned the most biomass above-ground (EMM = 0.47 g, SE = 0.03 g; Fig. 1d), followed by control (0.45 g, SE = 0.03 g), co-inoculated (0.40 g, SE = 0.03 g), and *C. geophilum* treatments (0.35 g, SE = 0.03 g). The model-based pairwise z-test with Benjamini-Hochberg correction revealed that *C. geophilum*-inoculated plants had significantly lower above-ground biomass than both control (*p* = 0.025) and *P. croceum*-inoculated plants (*p* = 0.025). For below-ground biomass, *P. croceum*-inoculated plants also showed the highest EMM (0.39 g, SE = 0.03 g), followed by co-inoculation (0.34 g, SE = 0.02 g), *C. geophilum* (0.32 g, SE = 0.02 g), and control treatments (0.27 g, SE = 0.03 g). However, none of the pairwise comparisons for root biomass were statistically significant (*p* = 0.42–0.89). The proportions of EMM of shoot and root dry weights of the different treatments indicate that in *C. geophilum*-inoculated plants, biomass allocation between shoots and roots was most even, whereas all other treatments showed higher allocation to shoots (Fig. S3).

## Time series analysis of developmental stages and growth cycles

At the end of the experiment, *P. croceum*-inoculated plants had completed the highest number of development stages (mean = 5.62, CV = 0.21; Fig. 2a–b), followed by control (5.05, CV = 0.33), co-inoculation (4.22, CV = 0.41), and *C. geophilum*-inoculated plants (3.04, CV = 0.49). During the selection step of the Bayesian regression model with a cumulative distribution family (Table S3), we chose the model with the highest Bayesian *R*^2^ of 0.96 and the lowest LOOIC of 1847.3. The model also showed sufficient R-hat (R-hat = 1.008) and bulk ESS values (ESS = 268). At the final time point (after 56 days), the model predicted the highest total number of development stages reached for *P. croceum*-inoculated plants during the experience (estimate = 6.65, SE = 0.25; Fig. 2c), followed by control (estimate = 5.95, SE = 0.36), co-inoculation (estimate = 5.04, SE = 0.35), and *C. geophilum* treatments (estimate = 4.02, SE = 0.38). Relative treatment effects showed that the *P. croceum* treatment had the strongest effect on development stage progression (relative estimate = 1.24; Fig. 2d), followed by *C. geophilum* (relative estimate = −1.05), co-inoculation (relative estimate = − 0.43), and control treatments (relative estimate = 0.27). Significant effects were observed for *P. croceum* (95% CI: 0.59 to 1.62) and *C. geophilum* treatments (95% CI: −1.47 to −1.05). Pairwise time series analysis showed significant differences in modelled growth developments between the *P.* croceum and co-inoculation treatments after four days, between *P. croceum* and *C. geophilum* treatments after six days, between control and *C. geophilum* treatments after 21 days, between *P. croceum* and control treatments after 33 days, between *C. geophilum* and co-inoculation treatments after 36 days, and between control and co-inoculation treatments after 45 days (Fig. 2e).

Pairwise Wilcoxon rank sum tests revealed no statistically significant differences in growth cycle durations between treatments. However, looking at individual development stages, throughout all treatments, plants remained the longest at bud rest stage A (mean = 17.25, CV = 0.66), followed by leaf maturation stage D (mean = 11.65, CV = 0.66), bud swelling stage B (mean = 8.51, CV = 1.04), and leaf expansion stage C (mean = 8.49, CV = 0.40; Fig. 2a, e). At specific development stages, Wilcoxon tests showed that *C. geophilum*-inoculated plants remained significantly longer at stage A than *P. croceum*- (*p* = 0.001) and control-inoculated plants (*p* = 0.012), while the co-inoculated plants remained significantly longer at stage A than the *P. croceum*-inoculated ones. At stage C, *C. geophilum*-inoculated plants remain significantly shorter than *P. croceum*- (*p* = 0.024) and co-inoculated plants (*p* = 0.012). There were no significant differences between treatments at stages B and D.

## Discussion

This study provides new insights into the differential effects of ECM fungi on biomass production and growth dynamics in the *Quercus robur* clone DF159, with a particular focus on the influence of these plant-fungus interactions on individual development stages during ERG. Our results demonstrate that fungal partners play a decisive role in shaping both overall plant productivity and the temporal dynamics of developmental stages, highlighting the species-specific effects of ECM-host interactions. Among treatments, *Piloderma croceum* significantly enhanced plant biomass production, despite showing low mycorrhization percentages (mean mycorrhization rate = 1,8%; 70% of plants colonized). These findings are consistent with previous studies suggesting that *P. croceum* improves nutrient mobilization at the rhizosphere level, allowing for increased growth without disrupting the ERG pattern (Herrmann et al. 2015; Tarkka et al. 2021).

In contrast, *C. geophilum*, despite its native association with oaks and high root colonization percentages (mean mycorrhization rate = 17%; 100% of plants colonized), led to the lowest biomass accumulation and the slowest shoot elongation phase, a critical and highly resource-demanding phase as described by Herrmann et al. (2015). This apparent paradox underlines the complexity of plant-fungal relationships (Ågren et al. 2019). While *C. geophilum* is known for its broad ecological niche and stress tolerance (Wang et al. 2025), yet in this controlled environment, its functional strategy may have limited its benefits to the host in terms of growth and biomass acquisition but reduced the duration of the critical phase of the shoot elongation while prolonging residence time at the steady stage A—characterized by net photosynthetic assimilation and the highest levels of non-structural carbohydrates during the growth cycle (Herrmann et al. 2015). This suggests that *C. geophilum*-inoculated plants either experienced developmental delays due to greater investment in mycorrhizal root tip formation or adopted a more resource-conserving growth mode. The latter may have led to an increased carbon-allocation to the root system (Hobbie and Colpaert 2004). Especially late successional plants have been shown to exhibit increased mycorrhizal responsiveness, characterized by resources allocation to mycorrhizal root tip formation at the expense of rapid biomass accumulation, suggesting a strategic shift towards mutualistic investment for improved stress resilience (Tang et al. 2024).

Based on our results showing that *C. geophilum*-inoculated plants complete significantly less growth stages as compared to the control plants in the same time window, our findings indicate that mycorrhization of *Q. robur* with *C. geophilum* might affect the duration of a growth cycle. However, due to the limited sample size, which prevents robust testing of treatment effects on the periodicity of growth rhythms, and the fact that the ERG is endogenously controlled, we cannot hypothesize that *C. geophilum* alters the internal clocks regulating the ERG (Herrmann et al. 2015). Similar to our findings of no increase in shoot biomass of host plants in association with *C. geophilum* as compared to non-mycorrhizal or other ECM, this pattern was previously described for *C. geophilum* interacting with Scots pine (*Pinus sylvestris;* Kipfer et al. 2012; Peter et al. 2016). However, significantly increased needle nitrogen content, photosynthesis, and water use efficiency for *C. geophilum*-inoculated plants as compared to non-mycorrhized ones was observed under well-watered conditions as well as signs of faster photosynthesis recovery after a drought event (Peter et al. 2016). This supports a possible beneficial effect of *C. geophilum* on robustness under environmental stress.

The co-inoculation treatment, which was included in this study to reflect the widely described phenomenon of trees being colonized by multiple ectomycorrhizal fungal species (Courty et al. 2008; Khokon et al. 2023; Lang et al. 2011; Smith et al. 2011), yielded intermediate biomass levels and development stage transitions. Notably, *C. geophilum* dominated root colonization (mean mycorrhization rate = 9.8%; 100% of trees colonized), outcompeting *P. croceum* in the root system (0% mycorrhization; Fig. S4). This supports prior assumptions of competitive advantages of *C. geophilum* in root colonization of temperate forest tree species (Looney et al. 2018). Considering that *C. geophilum* is described as a generalist ECM species (Peter et al. 2016; Wang et al. 2025) while *P. croceum* has a narrower ecological niche (Korkama et al. 2006; Van Schöll et al. 2006), our findings align with previous results stating that generalist ECM fungi have a competitive advantage over more specialized species (Lilleskov and Bruns 2003). While secondary metabolite biosynthesis genes have been shown to be more abundant in the *C. geophilum* genome than in those of ectomycorrhizal basidiomycetes (Peter et al. 2016), which may have implications for the interaction between *P. croceum* and *C. geophilum*, it appears that the dominance of *C. geophilum* did not translate into superior plant growth performance in the short term. This observation may be further explained by possible trade-offs between fungal competitive ability and symbiotic efficiency, characterized by higher colonization rates at the expense of enzymatic function, which has been shown for other ectomycorrhizal communities (Moeller and Peay 2016). The absence of *P. croceum* colonization in co-inoculated plants may explain why the growth-promoting effects observed in only *P. croceum*-inoculated plants were not replicated. Interestingly, *P. croceum* has previously been described as enhancing growth during the pre-symbiotic phase (Herrmann et al. 1998). The reduction in growth enhancement during the pre-symbiotic phase in this experiment emphasizes the importance to better understand co-inoculation effects. Our findings further reiterate the importance to study fungal community composition and interaction dynamics in shaping host outcomes in the light of ecological strategies of different fungal species (Anthony et al. 2024; Courty et al. 2010; Van Der Heijden et al. 2015).

A key novel insight from our study is the apparent modulation of stage-specific durations within the ERG by an ECM fungus. While previous studies have suggested that the ERG is internally regulated and largely buffered against changes in resource availability (Herrmann et al. 2015), our findings indicate that biotic interactions—specifically with the oak-native ECM fungus *C. geophilum*—can significantly alter plant development. This suggests that a certain degree of flexibility exists within plant developmental dynamics, enabling acclimatation through symbiotic relationships. Notably, *C. geophilum*-inoculated plants remained significantly longer at stage A, which corresponds to the steady state and the longest stage of a growth cycle also related to net photosynthetic carbon assimilation, that can be used for mycorrhizal root-tip formation. Our findings suggest that even in genetically uniform systems, development stage timing is not entirely immutable and may be fine-tuned by microbial partners. This supports emerging perspectives that endogenous plant rhythms, including ERG, offer high plasticity during plant responses to diverse trophic interactions by stage specific modulations in microbial signaling and other systemic cues (Fernández et al. 2024).

Overall, our findings underscore the functional diversity of ECM fungi and their capacity to modulate developmental timing and thereby biomass partitioning in a symbiont-specific manner. The clear differentiation in phenotypic outcomes between *P. croceum* and *C. geophilum* treatments illustrates how evolutionary history and ecological strategies of symbionts shape host physiological responses (Bouffaud et al. 2020). Importantly, this work contributes to a growing body of literature emphasizing the importance of integrating developmental dynamics into the study of plant-microbe interactions (Gaytán et al. 2022; Jumpponen and Jones 2009; Tarkka et al. 2013). In the context of climate change and increasing abiotic stress on *Q. robur* populations (Chen et al. 2025; Haneca et al. 2009), leveraging beneficial fungal partnerships, particularly those enhancing growth while maintaining developmental rhythms, may represent a promising avenue for forest management and restoration.

## Supplementary Information

Below is the link to the electronic supplementary material.

Supplementary Material 1

## Data Availability

Raw phenotypic data used in this study is available on the EnviDat repository (envidat.ch; https://www.doi.org/10.16904/envidat.649). In addition, raw phenotypic data and scripts used in the analysis are available on GitHub (https://github.com/nnamremmizxilef/2025_Mycorrhization_Experiment) and on the DataPLANT repository (nfdi4plants.org; https://git.nfdi4plants.org/phytoakmeter/mycorrhization/2025_mycorrhization_experiment).
