# Supplementary material for: An ectomycorrhizal fungus alters developmental progression during endogenous rhythmic growth in pedunculate oak
Source: Mycorrhiza. 2025 Oct 11;35(5):57. doi: 10.1007/s00572-025-01228-1 (PMC12515213; doi:10.1007/s00572-025-01228-1)
Supplement: Supplementary file 1 — Supplementary Material 1 [file 572_2025_1228_MOESM1_ESM.docx]

**Supplementary information**

**An ectomycorrhizal fungus alters the developmental stages of endogenous rhythmic growth in pedunculate oak**

Felix Zimmermann^1,2*^, Marie-Lara Bouffaud^3,4^, Sylvie Herrmann^3^, Marco Göttig^5^, René Graf^1^, Mika Tarkka^3,4^, Lars Opgenoorth^1,5^, Daniel Croll^2^, Martina Peter^1^ & Benjamin Dauphin^1*^

^1^ Biodiversity and Conservation Biology, Swiss Federal Research Institute WSL, Birmensdorf, Switzerland

^2^ Laboratory of Evolutionary Genetics, Institute of Biology, University of Neuchâtel, Neuchâtel, Switzerland

^3^ Department of Soil Ecology, Helmholtz Centre for Environmental Research - UFZ, Halle/Saale, Germany

^4^ German Centre for Integrative Biodiversity Research (iDiv) Halle-Jena-Leipzig, Leipzig, Germany

^5^ Plant Ecology and Geobotany, Institute of Biology, Philipps University Marburg, Marburg, Germany

* Corresponding authors: felix.zimmermann@wsl.ch, benjamin.dauphin@wsl.ch

**Figure S1** **Randomization of the oak plants for the experiment.** **a** Shows the randomization of plants by development stage. **b** Represents the randomization of plants by initial stem length. **c** Indicates the randomization of plants by divergence from the mean rooting date.

**

**Figure S2** **Spearman correlation coefficients of measured phenotypic traits.** Pairwise Spearman correlation coefficients (colored by rho value) for measured phenotypic traits during the destructive sampling. Descriptions of abbreviations can be found in Table S2. The upper triangle shows all pairwise correlations while the lower triangle shows only significant ones (*p* < 0.05).

**

**Figure S3** **Proportions of estimated marginal means of shoot and root dry weights.** Data is based on a generalized linear model where shoot and root dry weights are controlled for the total plant dry weight (plant part dry weight ~ treatment + plant dry weight). Different treatments are represented on the x-axis, proportions in percent on the y-axis, colors represent plant parts (shoot and roots).

**Figure S4** **Representative pictures of all treatments.** **a** Shows a control plant. **b** Shows a *Cenococcum geophilum*-inoculated plant. **c** Shows a *Piloderma croceum*-inoculated plant. **d** Shows a co-inoculated plant.

**

**Table S1** **Soil preparation for different treatments.** The vermiculite-peat inoculation cultures were mixed with equal parts (volume) of sterilized soil.

| Treatment | *Cenococcum geophilum*- inoculated vermiculite/peat 1:1 (v/v) | *Piloderma croceum*-inoculated vermiculite/peat 1:1 (v/v) | Non-inoculated vermiculite/peat 1:1 (v/v) |
| --- | --- | --- | --- |
| Control |  |  | x |
| *Cenococcum geophilum* | x |  |  |
| *Piloderma croceum* |  | x |  |
| Co-inoculation | x | x |  |

**Table S2** **Measured tree phenotypic traits.** List of traits measured during the destructive sampling, with the corresponding units and abbreviations used in the dataset.

| Trait | Unit | Abbreviation |
| --- | --- | --- |
| Tree Identifier | NA | ID |
| Treatment | NA | Treatment |
| Total Number of Shoot Flushes Reached | NA | nSF |
| Number of Leaves Second Newest Shoot Flush | NA | n_L_SF1 |
| Number of Leaves Newest Shoot Flush | NA | n_L_SF2 |
| Plant Alive? | NA | Alive |
| Plant Contaminated? | NA | Contaminated |
| Exclude Plant for Analyses? | NA | Exclude |
| Initial Stem Length at Plant Preparation | cm | Shoot_Length_Init |
| Stem Length at Sampling | cm | Shoot_Length |
| Stem Length Increase Throughout Experiment | cm | Delta_Length |
| Fresh Weight Total Plant | g | FW_Plant |
| Dry Weight Total Plant | g | DW_Plant |
| Fresh Weight Shoot | g | FW_Shoot |
| Dry Weight Shoot | g | DW_Shoot |
| Fresh Weight Stem | g | FW_Stem |
| Dry Weight Stem | g | DW_Stem |
| Fresh Weight Leaves Second Newest Shoot Flush | g | FW_L_SF1 |
| Dry Weight Leaves Second Newest Shoot Flush | g | DW_L_SF1 |
| Fresh Weight Leaves Newest Shoot Flush | g | FW_L_SF2 |
| Dry Weight Leaves Newest Shoot Flush | g | DW_L_SF2 |
| Fresh Weight Remaining Leaves | g | FW_L_Rest |
| Dry Weight Leaves Rest | g | DW_L_Rest |
| Fresh Weight Bud | g | FW_Bud |
| Dry Weight Bud | g | DW_Bud |
| Fresh Weight Root System | g | FW_Roots |
| Dry Weight Root System | g | DW_Roots |
| Fresh Weight Principal Roots | g | FW_PR |
| Dry Weight Principal Roots | g | DW_PR |
| Fresh Weight Lateral Roots | g | FW_LR |
| Dry Weight Lateral Roots | g | DW_LR |
| Leave Area Leaves Second Last Shoot Flush | cm^2^ | A_L_SF1 |
| Leave Area Leaves Last Shoot Flush | cm^2^ | A_L_SF2 |
| Number of *Cenococcum geophilum* Mycorrhized Root Tips | NA | N_Ceno |
| Number of *Piloderma croceum* Mycorrhized Root Tips | NA | N_Pilo |
| Number of Root Tips Mycorrhized by Other Species | NA | N_Other |
| Number of Non-Mycorrhized Root Tips | NA | N_No |
| Total Root Length | cm | Legth_Tot |
| Total Root Surface Area | cm^2^ | Area_Tot |
| Average Root Diameter | mm | Diameter_Avg |
| Total Root Volume | cm^3^ | Root_Vol |
| Number of Root Tips | NA | Tips |
| Number of Root Forks | NA | Forks |
| Number of Root Crossings | NA | Crossings |
| Length Fine Roots (0-1 mm Diameter) | cm | Length_Fine |
| Surface Area Fine Roots (0-1 mm Diameter) | cm^2^ | SA_Fine |
| Volume Fine Roots (0-1 mm Diameter) | cm^3^ | Vol_Fine |

**Table S3** **Bayesian regression model selection parameters.** Different parameters for identification of best model fit. A high Bayesian *R*^2^, R-hat values close to 1.0, a high bulk effective sample size (bulk ESS) and a lower leave-one-out information criterion (LOOIC) in comparison to other models indicate a good model fit. Best values per parameter are indicated in bold. The chosen model is shaded in green.

| Model | Bayesian R^2^ | Max. R-hat deviation from 1 | Min. Bulk Effective Sample Site | Leave-one-out Information Criterion |
| --- | --- | --- | --- | --- |
| Stages_Reached ~ Treatment * days_scaled + (1 \| ID) | 0.90 | 0.011 | 120 | 2914.6 |
| Stages_Reached ~ Treatment * days_scaled + (1 + days_scaled \| ID) | 0.95 | 0.010 | 215 | 2018.2 |
| Stages_Reached ~ Treatment * days_scaled + ar(time = days_scaled, gr = ID) + (1 \| ID) | 0.94 | 0.005 | 2941 | NA (NAs introduced during model convergence) |
| Stages_Reached ~ Treatment * days_scaled + ar(time = days_scaled, gr = ID) + (1 + days_scaled \| ID) | **0.96** | 0.007 | **3364** | 10172.4 |
| Stages_Reached ~ Treatment + s(days_scaled, by = Treatment) + (1 \| ID) | 0.92 | **0.003** | 171 | 2649.7 |
| Stages_Reached ~ Treatment + s(days_scaled, by = Treatment) + (1 + days_scaled\| ID) | **0.96** | 0.008 | 268 | **1847.3** |
